# Maternal and Early Life Iron Intake and Risk of Childhood Type 1 Diabetes: A Danish Case-Cohort Study

**DOI:** 10.3390/nu11040734

**Published:** 2019-03-29

**Authors:** Steffen Ullitz Thorsen, Thorhallur I. Halldorsson, Anne A. Bjerregaard, Sjurdur F. Olsen, Jannet Svensson

**Affiliations:** 1 Copenhagen Diabetes Research Center (CPH-DIRECT), Department of Paediatrics, Herlev Hospital, University of Copenhagen, Herlev Ringvej 75, 2730 Herlev, Denmark; Jannet.Svensson@regionh.dk; 2 Centre for Fetal Programming, Department of Epidemiology Research, Statens Serum Institut, 2300 Copenhagen S, Denmark; LUR@ssi.dk (T.I.H.); ANNE@ssi.dk (A.A.B.); SFO@ssi.dk (S.F.O.); 3 Unit for Nutrition Research, Faculty of Food Science and Nutrition, School of Health Sciences, University of Iceland, 101 Reykjavík, Iceland; 4 Department of Clinical Medicine, Faculty of Health and Medical Sciences, University of Copenhagen, Blegdamsvej 3B, 2200 Copenhagen N, Denmark

**Keywords:** diabetes mellitus, type 1, iron, pregnancy, fetal programming, infant, newborn

## Abstract

Background: Iron overload has been associated with diabetes. Studies on iron exposure during pregnancy and in early life and risk of childhood type 1 diabetes (T1D) are sparse. We investigated whether iron supplementation during pregnancy and early in life were associated with risk of childhood T1D. Methods: In a case-cohort design, we identified up to 257 children with T1D (prevalence 0.37%) from the Danish National Birth Cohort through linkage with the Danish Childhood Diabetes Register. The primary exposure was maternal pure iron supplementation (yes/no) during pregnancy as reported in interview two at 30 weeks of gestation (*n* = 68,497 with iron supplement data). We estimated hazard ratios (HRs) using weighted Cox regression adjusting for multiple confounders. We also examined if offspring supplementation during the first 18 months of life was associated with later risk of T1D. Results: Maternal iron supplementation was not associated with later risk of T1D in the offspring HR 1.05 (95% CI: 0.76–1.45). Offspring intake of iron droplets during the first 18 months of life was inversely associated with risk of T1D HR 0.74 (95% CI: 0.55–1.00) (p_trend_ = 0.03). Conclusions: Our large-scale prospective study demonstrated no harmful effects of iron supplementation during pregnancy and in early life in regard to later risk of childhood T1D in the offspring.

## 1. Introduction

Type 1 diabetes (T1D) is a chronic immune-mediated disease with a selective destruction of pancreatic β-cells leading to life-long insulin dependency and severe long-term complications [1,2]. T1D is triggered by both genetic and environmental factors [1]. Genetic susceptibility loci have been extensively identified [3], but only a few environmental factors have been proposed to associate with T1D [4]. The incidence of T1D is increasing at an annual rate of 3–5% in Denmark and many other countries [5,6], which suggest that yet unknown environmental factors play an important role in T1D etiopathology.

Iron, the most abundant metal in the human body, is an essential trace element for a plethora of cellular functions e.g., a normal maturation and function of the immune system [7,8]. Though, excess iron may be harmful e.g., due to formation of reactive oxygen species (ROS) by participating in the Fenton reaction [9]. Pancreatic β-cells possesses a classical iron metabolism [10] and are especially sensitive to ROS due to finite antioxidant defenses [11,12].

Iron deficiency and iron deficiency anemia are common conditions in pregnant women and during early childhood in both the European population and worldwide [13,14,15]. Iron requirement increases during pregnancy and therefore it is recommended that pregnant women take iron supplementations [13,14], and breastfed children are supplemented. However, in Western countries, such recommendations, on top of an already sufficient intake, could result in excess iron intake, in a subgroup of women. Further, use of several supplements or not following the recommended dosing can easily result in doses above the recommended upper intake levels [16,17]. Noteworthy, a study originated from the Danish National Birth Cohort (DNBC) reported that 37% of Danish pregnant women had an iron supplementation intake above the recommended 50–70 mg per day [16], which could lead to unfavorable iron concentrations in both the mother and child, but data regarding adverse effects of high maternal iron supplementation are still limited [18].

Though, much is still to be learned about the transport of iron from the maternal circulation through the placenta to the fetus, placental iron transport during pregnancy is mainly driven by the fetal need, but maternal iron deficiency or excess may compromise the fetal iron homeostasis [19,20,21]. It can be hypothesized that such extremes in maternal supply may have adverse consequences for the unborn child [22]. To our knowledge, only one study has examined the association between maternal iron status and intake and later risk of childhood T1D in the offspring. In the prospective Norwegian Mother and Child Cohort Study a positive association was found between maternal iron supplementation and risk of childhood T1D [23]. Further, only five relatively small-sized retrospective studies with moderate to high assessment quality have examined the association between early life iron exposure and later risk of childhood T1D, and results have been mixed [24,25].

Following up on the findings from the large Norwegian study we tested the hypothesis that there is an association between in utero and early life iron exposure and risk of childhood T1D, using pure iron supplementation data from the unique prospective DNBC [26] obtained through telephone interviews at 30 weeks of gestation, six and 18 months postpartum. Further, iron from both pure iron supplements and multivitamins were obtained from a 360-item food frequency questionnaire (FFQ) sent to the women at approximately 25 weeks of gestation [16].

## 2. Materials and Methods

### 2.1. Overview of Study Design

The study is a cohort study including maternal-child pairs from the DNBC [26] enrolling pregnant Danish women from 1996 until 2002. Altogether 91,745 mothers were recruited. Some women participated more than once leading to a total number of 101,042 pregnancies. Mothers were recruited at their first visit around 6–10 weeks of gestation at their general practitioner. All pregnant women residents in Denmark who were fluent in Danish were invited. Approximately 35% of all births in Denmark during the recruitment period were involved.

### 2.2. Study Sample and Identification of T1D

Approximately 260 controls per case exist in the cohort (sampling ratio 1:260). The proportion of any use of pure iron supplements during pregnancy in DNBC is 0.79. With 13,196 out of 63,931 women reporting use of pure iron supplements in pregnancy and the prevalence of offspring T1D being 0.37% we have a power of 80% to detect an absolute difference in risk of 1.6 when comparing users to non-users.

Patients were found through linkage with the Danish Childhood Diabetes Register (DanDiabKids), covering children aged 0–18 years diagnosed with T1D [27]. DanDiabKids was initiated in 1996 and is validated annually against the National Patient Discharge Register. DanDiabKids have nearly complete nationwide coverage and record high-quality prospective data on children with T1D. The time of clinical T1D diagnosis was set as the first day of insulin treatment in accordance with International Society for Pediatric and Adolescent Diabetes (ISPAD) guidelines. We included all children diagnosed with T1D who had available iron supplemental data for either one of the three time periods (pregnancy, and at six and 18 months of follow-up).

### 2.3. Exposure Assessment

#### 2.3.1. Maternal Iron Supplementation

Following up on the findings form the Norwegian Mother and Child Cohort study [23], our primary exposure was maternal use of pure iron supplements (supplements only containing iron) in early (<20 weeks) and late (≥20 weeks) pregnancy. Supplemental intake for these two time points was based on maternal report in telephone interviews conducted in week 30 of gestation and six months postpartum, respectively. In these interviews, the women were asked “Have you been taking iron pills during pregnancy?”. If they answered “yes” they were asked in which weeks of gestation they had been taking supplements, ranging from the beginning of pregnancy until the time of the 30-week interview; and the interview conducted at six months postpartum covered the remaining period of their pregnancy. In addition, we also assessed the total supplemental intake of iron as reported when the women filled out an FFQ in week 25 of gestation. The FFQ covered the supplemental intake of the previous four weeks. The women were asked to report all supplements they had been taken and for each label the amount of micronutrient, e.g., iron, and the daily dose [16].

In our study, the number of subjects available for analyses varied depending on the source of information. Information gained from interviews in week 30 of gestation and six months postpartum regarding use of pure iron supplements during pregnancy ranged from 63,931 to 64,456. Information on use of any iron containing supplements as reported by the mother in week 25 of gestation was available for 68,240.

#### 2.3.2. Infant Iron Supplementation

Iron supplement exposure during early life was extracted from the fourth telephone interview conducted at 18 months postpartum where the mother was asked if her child had received iron droplets (yes/no), and the duration of the supplementation (months). The women were asked “For how many months did he/she receive iron droplets on a regular basis?”, and the answered categories were as follows: i. under 1 month; ii. 1–2 months; iii. 3–6 months; and iv. over 6 months (https://www.dnbc.dk/-/media/arkiv/projekt-sites/dnbc/kodeboeger/interviews-1-4/code_book_interview_4.pdf?la=en).

#### 2.3.3. Other Variables

The following *a priori* covariates were included in the main adjusted models based on previous work and the literature: maternal age at delivery (continuous), pre-pregnancy body mass index (BMI) (<18.5, 18.5–24.9, 25–30, and ≥30 kg/m^2^), parity (nulliparous (yes/no)), smoking during pregnancy (never, occasional, and yes), parental socioeconomic status (high, medium, low, or student), duration of breastfeeding (0, 0–6, or 6+ months), caesarean section (yes/no), gestational age at delivery (continuous) (Table 1). The following covariates were considered for sensitivity analyses: maternal anaemia (yes/no), maternal celiac disease (yes/no), and maternal T1D (yes/no) (Table 1). Specification of included covariates in the adjusted models is presented in the footnotes of Table 2 and Table 3.

### 2.4. Statistical Approach

All details of the analysis plan were determined *a priori*. We used the mean and standard deviation (SD) to describe normally distributed continuous variables and percentages for dichotomous variables. Cox regression was used for examining associations between maternal or offspring intake of iron-containing supplements and later risk of developing T1D in the offspring. Offspring age from birth up to May 2016 was used as the underlying timescale—start of follow-up was 18 months in the offspring analyses (time of iron droplet data collection). The effect estimates were reported as hazard ratios (HR) with 95% CI. As women could enter the study repeatedly through different pregnancies, we used a robust sandwich covariance matrix estimate to account for interdependent observations. Level of significance was set as *p* = 0.05 (two-sided) and statistical analyses were performed using SAS version 9.2 (SAS Institute Inc., Cary, NC, USA). No adjustment for multiple testing was performed.

### 2.5. Ethics

The Regional Scientific Ethics Committee for the municipalities of Copenhagen and Frederiksberg approved all DNBC study protocols. Approval from the Danish Data Protection Agency was also obtained. All procedures were in accordance with the Declaration of Helsinki. All women provided written informed consent.

## 3. Results

### 3.1. Basic Characteristics

The characteristics of the study participants are presented in Table 1. We confirm a higher percentage with high socio-economic status among those who take iron during pregnancy and give iron supplementation to their child. Whereas maternal celiac disease and anemia seems to influence the likelihood of supplementation during early pregnancy, but not supplementation to the child.

The median age at T1D diagnosis was 9.8 years (range 0.9–16.9), and the median follow-up time for the cohort sample was 15.6 years (range 13.0–18.6). Maternal intake of any iron supplement was below 40 mg/daily and above 80 mg/daily in 38.2% and 4.6% of the women, respectively. The proportion of women reporting to take pure iron supplement during early (<20 weeks) and late (≥20 weeks) pregnancy was 17.3% and 75,6%, respectively. The proportion of children receiving iron droplets during the first 18 months of life was 53.2%—the majority of these children (92.3%) were only supplemented with iron droplets for six months or less.

### 3.2. Maternal Iron Supplementation

Our primary adjusted analysis demonstrated that there was no association between maternal pure iron supplementation during pregnancy and later risk of childhood T1D in the offspring HR 1.05 (95% CI: 0.76–1.45). Further, maternal pure iron supplementation was not associated with risk of T1D in the offspring prior to or after gestational week 20: HR 0.82 (95% CI: 0.57–1.17) and HR 1.13 (95% CI: 0.83–1.53), respectively (Table 2). Further, no association was found when categorizing maternal iron intake from any supplement in mid-pregnancy and later risk of T1D in the offspring (p_trend_ = 0.82) (Table 2).

### 3.3. Infant Iron Supplementation

**Figure 1:**
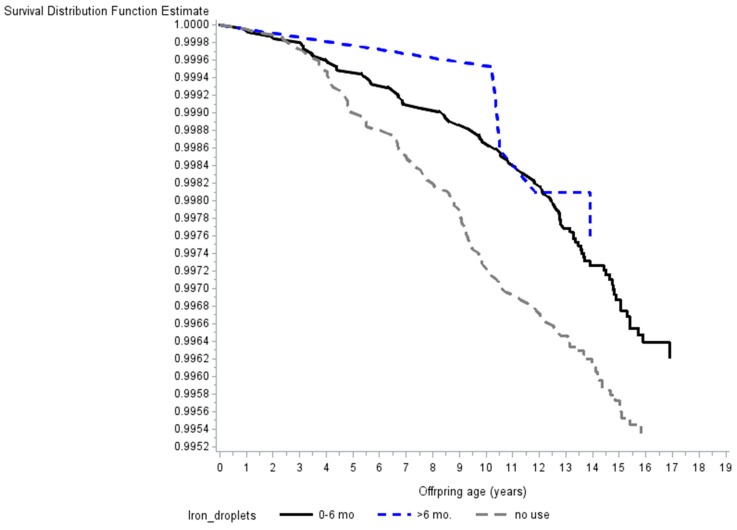
Survival curve illustrating the inverse association between early life supplementation with iron droplets and the risk of childhood type 1 diabetes. *p*-values for trend and effect are seen in Table 3. Note that the y-axis does not begin at zero.

We found an inverse association between iron supplementation during the first 18 months of life and risk of childhood T1D HR 0.74 (95% CI: 0.55–1.00) (p_trend_ = 0.03) (Table 3) (Figure 1).

### 3.4. Sensitivity Analyses

Our results were essentially unchanged when additional adjustment for maternal anaemia, maternal celiac disease, and maternal T1D were performed (Table 2 and Table 3).

When we start follow-up at 30 months of age (one year after information of iron droplets was collected) no differences are seen in our results regarding offspring risk of T1D.

## 4. Discussion

In this study, which utilizes one of the world’s largest cohorts of pregnant women, we show that maternal iron intake through supplementation is not associated with later risk of childhood T1D in the offspring, but iron supplementation in early childhood may be protective against childhood T1D.

### 4.1. Comparison with Other Studies

#### 4.1.1. Studies Regarding Maternal Iron Intake and Later Risk of Childhood T1D

To our knowledge only the prospective Norwegian Mother and Child Cohort Study has examined the relationship between maternal iron supplementation and status, and later risk of T1D in the offspring [23]. They found a positive association between any maternal iron supplementation and childhood T1D (adjusted HR 1.33 (95% CI: 1.06–1.67)), when only supplements containing pure iron or iron in combination with other micronutrients were examined the results were no longer statistically significant (adjusted HR 1.71 (95% CI: 0.83–3.53)) and (adjusted HR 1.60 (95% CI: 0.95–2.69)), respectively. The Norwegian study found no association between maternal intake of iron from food and later risk of T1D in the offspring.

#### 4.1.2. Iron Supplementation Guidelines in Denmark and Norway during the Recruitment Period

*Størdal and colleagues’* states that iron supplementation of pregnant women was during the first half of their recruitment period (2000–2005) based on national guidelines concerning ferritin measurements. Revised guidelines did not recommend serum ferritin measurements, but rather recommended haemoglobin measurement at week 30. However, analyses revealed iron supplementation was given as a broad recommendation (40 mg/d from gestational week 18–20) during the whole study period, and to a lesser extend guided by indices of body iron stores [23]. During the DNBC recruitment period all Danish pregnant women were advised to take a daily supplement of 50–70 mg iron from week 20 of gestation until delivery [28]. In the DNBC study, 37% of Danish pregnant women had an iron supplementation intake above the recommended 50–70 mg per day, due to inappropriate product formulations on the Danish market [16]. This indicates that Danish pregnant women, during the overlapping recruitment periods, had a higher supplemental iron exposure compared to the Norwegian women, but still we did not find an association between supplemental iron exposure and risk of T1D in the offspring. Iron supplements were used at some point in 64% of the women in the Norwegian study versus 79% of the Danish women that took pure iron supplements at some point during pregnancy [23].

#### 4.1.3. Studies Regarding Infant Iron Intake and Later Risk of Childhood T1D

The Norwegian Mother and Child Cohort Study found no association between early life (<18 months of life) iron supplementation and risk of childhood T1D (adjusted HR 1.22 (95% CI: 0.78–1.90)). Infant iron supplementation in Norway was not common practice during the study period. At six and 18 months only 4.3% and 1.4% of the Norwegian infants received an iron supplement, respectively, which leaves this study with insufficient power to detect a possible effect [23]. In the DNBC study 49.1% of the infants received iron droplets during a period of one to six months, but only 4.1% received iron droplets longer than six months (Table 3). During the DNBC study recruitment period the Danish Health Authority recommended that children were iron supplemented between six and 12 months.

In addition, only five retrospective studies have looked at iron exposure before the age of 16 years and risk of developing childhood T1D [25,29,30,31,32]. The results are inconsistent but there are important methodological differences and limitations to take into account. A total of three studies, one retrospective and two case-control studies, focused on the content and amount of trace metals, including iron, in drinking water at the time of T1D diagnosis. None of these studies found statistically significant associations, but the study by Samuelson and colleagues suggested that a high iron content in the drinking water increases the risk of childhood T1D odds ratio (OR) 1.56 (95% CI: 0.99–2.44). These studies reported iron concentrations in drinking water of 0.01–0.08 mg/L, which contributes to a negligible iron intake compared to iron gained from supplementation and diet [33]. Moreover, iron exposure around time of childhood T1D diagnosis represents another “window of vulnerability” compared to the present study’s exposure interval i.e., in utero to 18 months of age. Further, one case-control study found that the odds ratio for one SD increase in iron intake (from infant formulas and breast milk) was 2.01 (95% CI: 1.18–3.41) during the first four months of life [32]. Iron exposure was quantified from a self-administered questionnaire filled out by parents of children with T1D under the age of 10 years. Children were diagnosed with T1D between one and six years of age. Using self-reported questionnaires on dietary recall over a long duration may introduce differential misclassification of exposure i.e., mothers of control subjects may under-report exposure and mothers of case subjects may over-report, which in both situations leads to overestimation of the association [34]. Lastly, a Danish case-control study, originated from our research group, quantified neonatal whole blood (WB-Iron) iron content. For each doubling in WB-Iron content the odds of developing childhood T1D was 2.55 (95% CI: 1.04–6.24). This study only had one biological measurement and no information on iron homeostasis genetics, but a direct quantification of total iron content using a valid method seems superior to questionnaire data on iron intake, due to variation in bioavailability of iron from both the food source and supplements [35,36], competing mechanisms at the di-metal luminal transporters, and genetic factors regulating the iron transport from the intestinal lumen to the circulation [24,37] However, the measured WB-Iron content at birth is likely to reflect the intrauterine exposure and not early life supplementation.

Our finding that supplemental iron intake during pregnancy is not associated with childhood T1D in the offspring, may primarily be due to: i. a tight placental iron regulation that is affected by dietary/supplemental iron intake and the iron metabolism genetic make-up in both the mother and the fetus [38]; ii. difference in intestinal absorption depending on supplemental-regime e.g., magnitude of hepcidin counterbalance when iron supplements are taken consecutively versus alternately [36]; and iii. no effect of iron on risk of childhood T1D. Regarding the placental iron regulation, neonates born by women that had been supplemented with 66 mg elemental iron daily during pregnancy had s-ferritin concentrations that were higher compared to neonates born of women that did not receive an iron supplement during pregnancy (155 versus 118 microgram/L). Neonates born by women with s-ferritin below 13.6 microgram/L also have lower levels of s-ferritin [19]. S-ferritin < 20 microgram/L indicates exhausted iron stores [39]. The Norwegian Mother and Child Cohort Study only found a suggestive non-significant positive association between s-ferritin in cord blood and later risk of childhood T1D (adjusted odds ratio 1.05 (95% CI: 0.99–1.13) per 50 mg/L increase) [23]. The 5th–95th percentile reference interval for cord s-ferritin was 40–468 microgram/L in Norway [40]. Hay et al. states that cord s-ferritin is a strong predictor of iron status the first two years of life [40].

### 4.2. Strengths and Weaknesses

Our study benefits from several strengths: (i) a relative large-scale study with a prospective design; (ii) we were able to obtain information on iron intake from both pregnancy and early childhood; and (iii) multiple possible confounders were included in our analyses.

Some limitation should also be considered: (i) we have no genetic data regarding iron homeostasis; (ii) using iron intake as *a proxy* for body iron status is inferior to direct quantification [15], but the fact that iron supplementation is associated with iron-status biomarkers in both pediatric and adult populations [41,42], and iron requirements fall within a relatively narrow band, with low and high intakes being harmful, makes data on iron intake, especially from large prospective cohort, a valuable source for gaining new insight within this field of study; (iii) participants in the DNBC study may not be representative of the general population of pregnant women in Denmark (e.g., they may have a healthier lifestyle and be better educated), but this does not necessarily confound exposure-outcome associations [43]; (iv) users of dietary supplements in general have a healthier diet than non-users, and individuals who take dietary supplements are the most unlikely to need them [16], which, in the DNBC study, results in ~37% of the women being exposed to iron supplements intakes that exceeds the recommended daily dose of 50–70 mg during pregnancy; (v) our results may be generalizable to other European and European origin populations, but may not be generalizable to populations with higher prevalence of iron deficiency and iron deficiency anemia; (vi) very few women did not take iron supplementation in Denmark decreasing the power leaving the 33% increased risk found in the Norwegian study within our 95% CI; (vii) we did not have any data on development of persistent islet autoantibodies in the children, prior to T1D diagnosis, and were therefore not able to examine the effect of maternal and offspring iron supplementation on the risk of initiation (seroconversion) and/or acceleration (epitope spreading) of islet autoimmunity. Our start of follow-up was 18 months, in the offspring analyses, excluding children diagnosed before this age (1.56%) and no change in results were seen when follow-up was postponed one year (30 months of age); (viii) potential micronutrient-micronutrient interactions regarding association with childhood T1D could not be well-examined in this study. Though, there is no strong evidence in the literature that iron supplementation would e.g., affect zinc status negatively or vice versa or that low zinc status could increase risk of T1D [44,45,46]; and (ix) our results may be influenced by residual confounding.

### 4.3. Implications and Future Perspective

In contrast with previous reports we found no indication that maternal use of iron supplements during pregnancy is harmful with respect to later risk of developing childhood T1D in the offspring. In addition, infant use of iron droplets during the first 18 months of life, was if anything, protective with respect to later T1D risk, but these findings need to be confirmed before strong conclusions can be reached. Future prospective studies need to be large-scale and must preferable include sensitive iron status markers and a broad-range of iron homeostasis single nucleotide polymorphisms for a thorough understanding of complex iron-genotype interactions, which could shed light on possible at-risk/protected groups.

## 5. Conclusions

Our large-scale prospective study demonstrated no harmful effects of iron supplementation during pregnancy and in early life in regard to later risk of childhood T1D in the offspring.

## Figures and Tables

**Table 1:** Maternal characteristics in relation to maternal use of pure iron supplements and offspring intake of iron droplets during the first 18 months of infancy.

	All	Maternal Use of Iron Supplements			Offspring Use of Iron Droplets	
		No Use (*n* = 15,071)	Early Use (*n* = 11,092)	Late Use (*n* = 48,318)	No (*n* = 24,281)	Yes (*n* = 27,595)
Maternal age (years)	30.4 (4.2)	30.7 (4.4)	30.7 (4.2)	30.3 (4.1)	30.5 (4.2)	30.6 (4.1)
Pre-pregnancy BMI (kg/m^2^)						
% underweight (<18.5)	4.3%	4.1%	5.4%	4.2%	3.9%	4.2%
% normal weight (18.5–25)	68.2%	66.1%	70.5%	68.8%	66.4%	69.7%
% overweight (25–30)	19.6%	20.6%	17.8%	19.4%	20.9%	18.8%
% obese (>30)	7.9%	9.2%	6.4%	7.6%	8.8%	7.3%
Maternal Smoking, %	24.5%	27.2%	25.5%	23.4%	26.0%	21.5%
Nulliparous, %	48.4%	39.5%	41.3%	51.5%	45.5%	49.8%
Socio-economic status						
High, %	55.3%	52.7%	54.9%	56.3%	52.6%	58.9%
Medium, %	27.2%	27.9%	28.0%	27.1%	29.8%	25.4%
Low, %	12.2%	14.3%	12.7%	11.3%	13.0%	10.3%
Students, %	5.3%	5.1%	4.5%	5.3%	4.6%	5.3%
Breastfeeding, %						
No	11.1%	11.6%	11.1%	10.8%	13.2%	6.3%
1–6 months	28.4%	28.9%	28.8%	28.1%	33.3%	17.5%
6+ months	60.6%	59.5%	60.1%	61.1%	53.5%	76.2%
Gestational age at delivery (days)	280.2 (12.4)	280.8 (11.5)	280.9 (21.9)	280.8 (11.2)	281.1 (10.8)	279.8 (13.0)
Cesarean section, %	15.3%	14.1%	13.8%	15.2%	15.1%	15.1%
Maternal celiac disease (*n*,%)	172, 0.25%	36, 0.24%	45, 0.41%	122, 0.25%	57, 0.23%	64, 0.24%
Maternal type 1 diabetes (*n*,%)	332, 0.49%	77, 0.51%	44, 0.40%	232, 0.48%	114, 0.47%	137, 0.53%
Maternal anemia (*n*,%)	1916, 3.5%	427, 3.6%	606, 7.0%	1374, 3.5%	732, 3.6%	804, 3.5%

Early use = before gestational week 20; late use = after gestational week 20; Body Mass Index, BMI.

**Table 2:** Associations between maternal intake of pure iron supplements during pregnancy and total intake of iron from supplements in relation to offspring risk of type 1 diabetes.

No. Cases (%)/N		Unadjusted	Adjusted 1 ^1^	Adjusted 2 ^2^
**Any use of pure iron supplements** (*n* = **63,931**)				
No	**48 (0.36%)**/13,196	1.00 (reference)	1.00 (reference)	1.00 (reference)
Yes	**190 (0.37%)**/50,735	1.06 (0.77, 1.46)	1.05 (0.76, 1.45)	1.05 (0.77, 1.45)
**Early use of pure iron supplements in gestational week 1 to 19** (*n* = **63,931**)				
No	**203 (0.38%)**/52,834	1.00 (reference)	1.00 (reference)	1.00 (reference)
Yes–early	**35 (0.32%)**/11,097	0.81 (0.57, 1.16)	0.82 (0.57, 1.17)	0.81 (0.57, 1.16)
**Late use of pure iron supplements in gestational week 20 to 40** (*n* = **63,931**)				
No	**54 (0.35%)**/15,613	1.00 (reference)	1.00 (reference)	1.00 (reference)
Yes–late	**184 (0.38%)**/48,318	1.14 (0.84, 1.54)	1.13 (0.83, 1.53)	1.13 (0.83, 1.54)
**Total supplemental iron intake as reported in week 25 of gestation** (*n* = **68,240**)				
0 mg/day	**29 (0.42%)**/6880	1.00 (reference)	1.00 (reference)	1.00 (reference)
>0–20 mg/day	**12 (0.28%)**/4308	0.66 (0.34, 1.29)	0.67 (0.34, 1.32)	0.66 (0.34, 1.31)
>20–40 mg/day	**51 (0.34%)**/14,934	0.81 (0.51, 1.27)	0.80 (0.50, 1.26)	0.79 (0.50, 1.25)
>40–60 mg/day	**117 (0.40%)**/29,539	0.95 (0.63, 1.43)	0.94 (0.62, 1.43)	0.94 (0.86, 1.41)
>60–80 mg/day	**37 (0.39%)**/9431	0.94 (0.58, 1.53)	0.94 (0.58, 1.53)	0.93 (0.57 1.52)
>80 mg/day	**11 (0.35%)**/3148	0.81 (0.41, 1.63)	0.81 (41, 1.63)	0.80 (0.40, 1.60)
*p*-value for trend		0.80	0.82	0.84

Number and percentage of cases are depicted in bold. ^1^ Adjusted for parental socio-economic status, mode of delivery, pre-pregnancy BMI, age, smoking status, parity, gestational age, maternal age, and breastfeeding. ^2^ same as model 1 but in addition adjustments are made for maternal celiac disease, maternal type 1 diabetes and maternal anemia.

**Table 3:** Associations between offspring intake of iron droplets during first 18 months of life in relation to later risk of type 1 diabetes.

No. Cases (%)/N		Unadjusted	Adjusted 1 ^1^	Adjusted 2 ^2^
**Offspring use of iron droplets reported at 18 months** (*n* = **51,859**)				
No	**104 (0.43%)**/24,272	1.00 (reference)	1.00 (reference)	1.00 (reference)
Yes	**87 (0.32%)**/27,587	0.73 (0.55, 0.97)	0.74 (0.55, 1.00)	0.73 (0.55, 0.99)
No	**104 (0.43%)**/24,272	1.00 (reference)	1.00 (reference)	1.00 (reference)
1–6 months	**82 (0.32%)**/25,483	0.75 (0.56, 1.00)	0.76 (0.56, 1.02)	0.75 (0.55, 1.01)
>6 months	**5 (0.24%)**/2104	0.56 (0.28, 1.37)	0.55 (0.23, 1.35)	0.56 (0.23, 1.36)
*p*-value for trend ^3^		0.03	0.03	0.03
*p*-value for effect ^4^		0.08	0.10	0.09

Number and percentage of cases are depicted in bold. ^1^ Adjusted for parental socio-economic status, mode of delivery, pre-pregnancy BMI, age, smoking status, parity, gestational age, maternal age, and breastfeeding. ^2^ Same as model 1 but in addition adjustments are made for maternal celiac disease, maternal type I diabetes and maternal anemia. ^3^ Chi-square-test is used to test for a linear dose-response—the iron variable is included in the regression model as a continuous (three values) variable. ^4^ Chi-square-test is used to test the null hypothesis that all three groups are equal.
